# Diversity of *Borrelia burgdorferi* sensu lato in ticks and small mammals from different habitats

**DOI:** 10.1186/s13071-022-05326-3

**Published:** 2022-06-07

**Authors:** Nina Król, Anna Obiegala, Christian Imholt, Charlotte Arz, Elisabeth Schmidt, Kathrin Jeske, Rainer Günter Ulrich, Zaida Rentería‑Solís, Jens Jacob, Martin Pfeffer

**Affiliations:** 1 Institute of Animal Hygiene and Veterinary Public Health, University of Leipzig, An den Tierkliniken 1, 04103 Leipzig, Germany; 2 Institute of Novel and Emerging Infectious Diseases, Friedrich-Loeffler-Institut, Südufer 10, 17493 Greifswald, Insel Riems Germany; 3 Institute for Parasitology, Centre for Infectious Diseases, Faculty of Veterinary Medicine, University of Leipzig, An den Tierkliniken 35, 04103 Leipzig, Germany; 4 Institute for Plant Protection in Horticulture and Forests, Julius Kühn-Institute, Toppheideweg 88, 48161 Münster, Germany

**Keywords:** Germany, Grassland, Forest, Sequence type, Multilocus sequence typing, *Clethrionomys*, *Microtus*, *Apodemus*, *Sorex*, *Ixodes*

## Abstract

**Background:**

Ixodid ticks are important vectors for zoonotic pathogens, with *Ixodes ricinus* being the most important in Europe. Rodents are hosts of immature life stages of *I. ricinus* ticks and are considered main reservoirs for tick-borne pathogens, e.g. *Borrelia burgdorferi*. The aim of this study was to analyse the prevalence as well as genospecies and sequence type (ST) diversity of *Borrelia burgdorferi* sensu lato in ticks and small mammals from central Germany and to elaborate on the influence of environmental and/or individual host and vector factors on *Borrelia* prevalence.

**Methods:**

After species identification, 1167 small mammal skin samples and 1094 ticks from vegetation were screened by *B. burgdorferi* sensu lato real-time polymerase chain reaction, and positive samples were characterized by multilocus sequence typing. Generalized linear (mixed) models were used to estimate how seasonality, small mammal species/tick life stage and habitat affect individual infection status.

**Results:**

In total, 10 small mammal species and three tick species, *Ixodes ricinus*, *Ixodes inopinatus* (both considered members of the *I. ricinus* complex) and *Dermacentor reticulatus*, were investigated. *Borrelia* DNA was detected in eight host species, i.e. the striped field mouse (*Apodemus agrarius*), the yellow-necked field mouse (*Apodemus flavicollis*), the wood mouse (*Apodemus sylvaticus*), the water vole (*Arvicola amphibius*), the bank vole (*Clethrionomys glareolus*), the field vole (*Microtus agrestis*), the common vole (*Microtus arvalis*), and the common shrew (*Sorex araneus*). Two species were *Borrelia *negative, the greater white-toothed shrew (*Crocidura russula*) and the pygmy shrew (*Sorex minutus*). The average prevalence was 6.2%, with two genospecies detected, *Borrelia afzelii* and *Borrelia garinii*, and at least three STs that had not been previously reported in small mammals. *Borrelia* prevalence in small mammals did not differ between seasons. Six genospecies of *Borrelia—Borrelia afzelii*, *Borrelia valaisiana*, *Borrelia garinii*, *Borrelia lusitaniae*, *Borrelia spielmanii*, and *Borrelia burgdorferi* sensu stricto—and 25 STs of *Borrelia*, of which 12 have not been previously described at all and five have not been previously reported in Germany, were detected in 13% of *I. ricinus* complex ticks. Prevalence was highest in adult females (25.3%) and lowest in nymphs (11.4%). Prevalence was significantly higher in ticks from grassland (16.8%) compared to forests (11.4%).

**Conclusions:**

The high level of small mammal diversity in this region of Germany seems to be reflected in a wide variety of genospecies and STs of *B. burgdorferi*.

**Graphical abstract:**

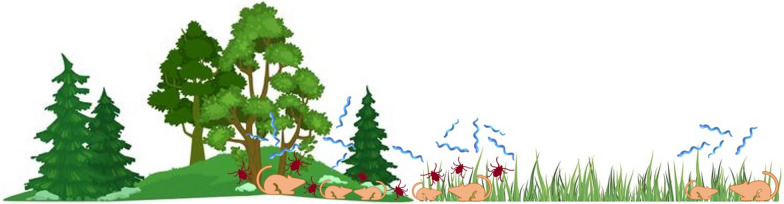

**Supplementary Information:**

The online version contains supplementary material available at 10.1186/s13071-022-05326-3.

## Background

Hard ticks (Ixodidae) are important vectors of zoonotic pathogens, including bacteria, protozoan parasites, and viruses. The most common tick species in Europe is the castor bean tick, *Ixodes ricinus*. Due to its wide spectrum of hosts and its environmental plasticity, it occurs in several types of habitats and it is important for the transmission of several zoonotic pathogens [1]. Its immature life stages (larvae and nymphs) parasitize mostly small mammals and birds. The second most common tick species in temperate Europe is the ornate cow tick, *Dermacentor reticulatus*, whose juvenile developmental stages feed mostly on small mammals [2, 3]. Therefore, small mammals, predominantly rodents, are essential for the maintenance and distribution of ticks and tick-borne pathogens [4, 5].

In Europe, the most frequently reported tick-borne disease is Lyme disease, which is caused by *Borrelia burgdorferi* sensu lato (s.l.), a complex of gram-negative spirochetes. *Borrelia burgdorferi* s.l. is transmitted mostly by *I. ricinus* ticks, but has also occasionally been found in *D. reticulatus* [6–8].

To date, over 20 genospecies of the *B. burgdorferi* s.l. complex have been identified, with 11 of them occurring throughout Europe [9]: *Borrelia afzelii*, *Borrelia bavariensis*, *Borrelia bissettiae* (previously known as *Borrelia bissettii* [10]), *Borrelia burgdorferi* sensu stricto (s.s.), *Borrelia carolinensis*, *Borrelia finlandensis*, *Borrelia garinii*, *Borrelia kurtenbachii*, *Borrelia lusitaniae*, *Borrelia spielmanii*, and *Borrelia valaisiana*. Most of them are known to be pathogenic for humans. *I. ricinus* is the main vector for the 11 European genospecies of *Borrelia*, but the reservoir host species range is much more diverse and genospecies specific [9, 11]. More than 40 different vertebrate species, including reptiles, birds, and mammals, may serve as reservoir hosts of *Borrelia* [4, 5, 12–15]. The adaptation of *B. burgdorferi* s.l. species to different reservoir hosts is an important driver for their spatial distribution and diversification in ecosystems [16]. In a complex multi-host system, *Borrelia burgdorferi* s.l. circulation is difficult to monitor and forecast in the respective ecosystems [17]. According to the biodiversity-driven dilution effect hypotheses, the balance between competent (reservoir) and incompetent (dilution, rescue) hosts is crucial for the risk of infection [18–20]. Feeding on a dilution host species may clear a pre-existing *Borrelia* infection in *I. ricinus* [21, 22]. Clearance might therefore occur more often in communities dominated by incompetent hosts. The rescue hosts mostly support the life cycle of ticks, but are capable of maintaining disease risk when reservoir host density is low.

Genotyping of *Borrelia* spp. is a helpful tool for the description of epidemiological and ecological patterns. *Borrelia* genospecies are usually further categorized into sequence types (STs) by multilocus sequence typing (MLST), which is based on eight housekeeping genes: *nifS*, *pyrG*, *clpX*, *pepX*, *uvrA*, *rplB*, *cplA*, and *recG*. These genes are characterized by slow evolutionary rates and an almost neutral variation resulting in robust phylogenetic relationships and the avoidance of potentially skewed clustering in single-locus sequence analyses [16, 23]. Factors such as habitat, geographical region, host, and vector species may have an impact on the genetic variability of certain *Borrelia* genospecies. The mechanisms of rapid adaptation through alterations in gene expression allow the spirochetes to adjust and survive in different host species [24–26]. However, there is scarce information on the variation of STs in mammals and ticks when considered on a small geographical scale, and the habitat associations of certain STs.

The aim of this study was to determine the prevalence and (geno)diversity of *Borrelia burgdorferi* s.l. in small mammals (21 sites) and ticks (17 sites) from different forest and grassland sites in central Germany, and to elaborate on the effects of habitat, season, and small mammal species richness on *Borrelia* prevalence.

## Methods

### Study area

**Fig. 1:**
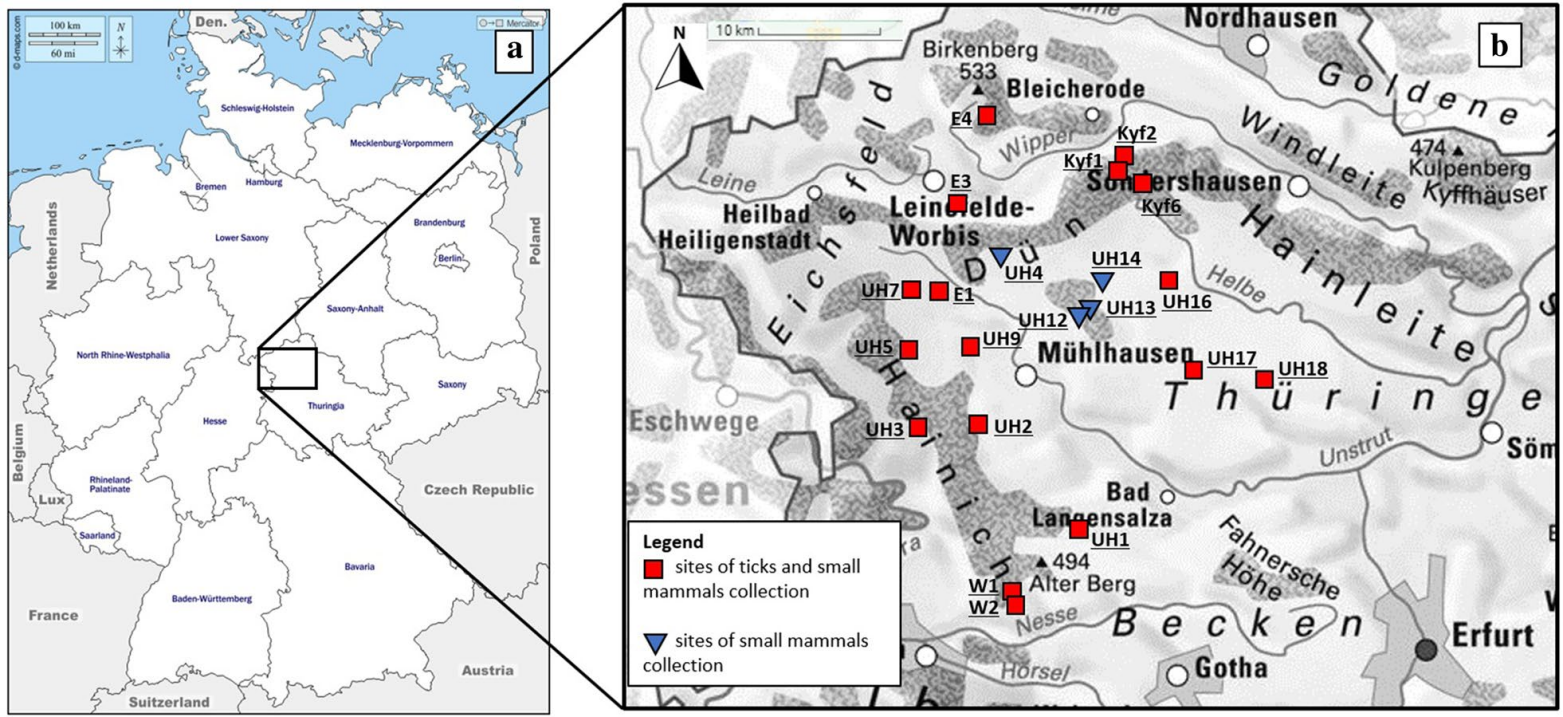
**a** Location of the study area (*inset*) in Germany (https://d-maps.com/carte.php?num_car=4692&lang=en), and **b** locations of the 21 collection sites within the study area (source en.wikipedia.org, with the authors’ own modifications)

The study sites (Fig. 1) were located in the Hainich Dün region surrounding the Hainich National Park, which is one of the largest continuous deciduous forests in Germany. Outside the national park the grasslands are grazed extensively, primarily by sheep, and the forests are managed by selection cutting. Due to its large area, which is protected and under a balanced system of local management, the Hainich Dün region has a high animal and plant biodiversity (https://www.biodiversity-exploratories.de/en/regions/hainich-duen/).

### Small mammal and tick collection

Skin samples from small mammals trapped using snap traps at 21 sites in central Germany were available from a former study [27, 28] (Fig. 1). Trapping took place in spring, summer and autumn in 2017, 2018 and 2019. For this study, 1167 out of 1945 samples from all 21 sites, comprising 168 spring samples (range 24–146) (2018 and 2019) and 999 summer samples (range 72–639) (2017, 2018 and 2019), which represented 60% of the original material, were randomly selected.

Ticks were collected from vegetation at 17 of the 21 sites by the flagging method [28] once per season along 100-m^2^ transects in spring, summer and fall 2018 and 2019. The number of collection sites for ticks was limited to 17 due to financial restrictions. The ticks were stored in 70% ethanol and the small mammal skin samples in a freezer at − 20 °C until further examination.

**Fig. 2:**
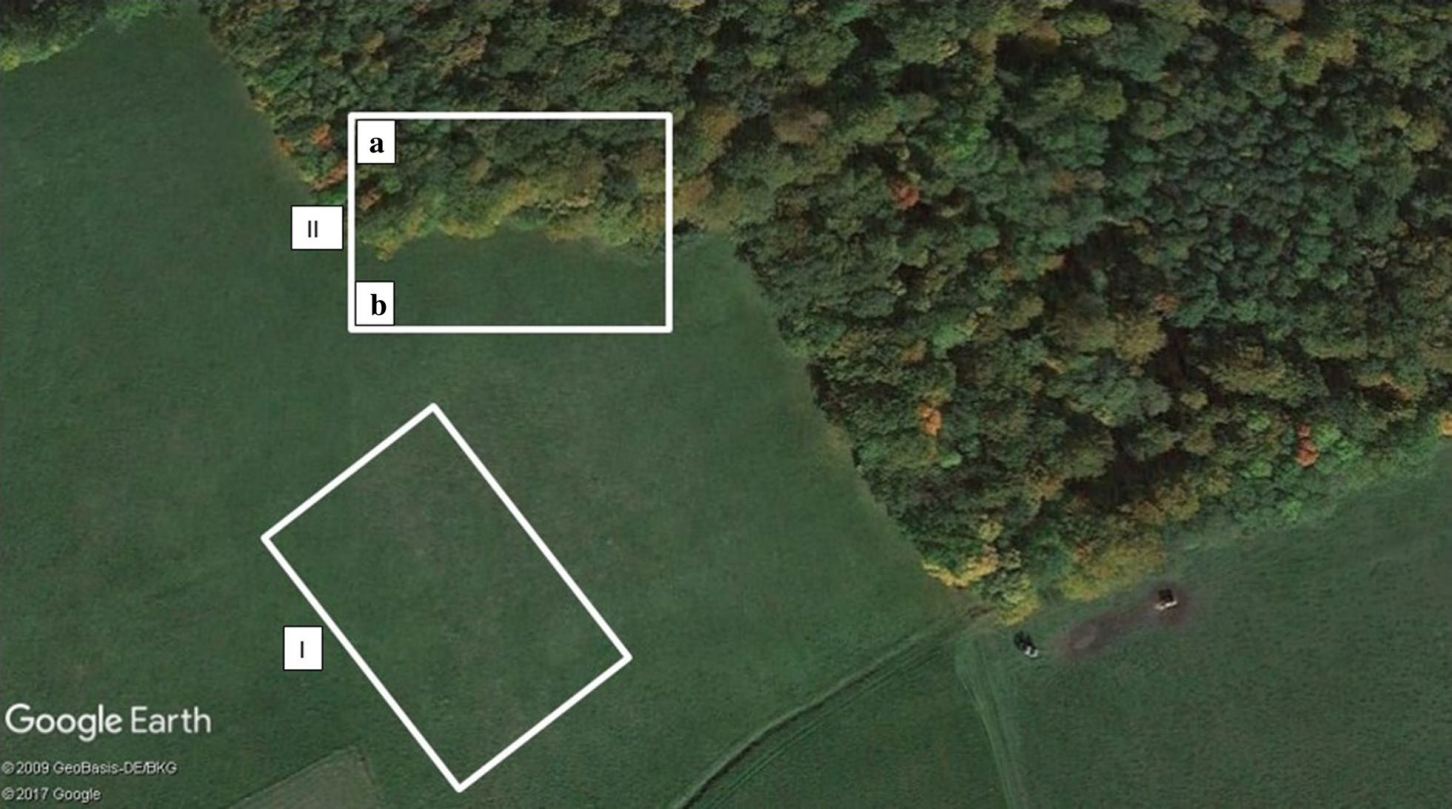
Photograph of study sites showing paired forest and grassland habitats [grassland habitat of small mammals (I), forest habitat of small mammals (II), forest habitat of ticks (IIa), grassland habitat of ticks (IIb)] (source Google Earth with the authors’ own modifications)

At each site, small mammal trapping was performed in grasslands (I) and forests (II), and tick collection was conducted within the ecotone at the edge of forests, which comprised woodland and adjacent grassland (Fig. 2). Ticks were not collected from the grassland habitat where the small mammals were trapped (I), but were collected from the grassland (IIb) and woodland areas (IIa) of the forest habitat (II). Thus, grassland habitat of ticks refers to the grassland part of the forest habitat (IIb) and forest habitat of ticks refers to the woodland part of the forest habitat (IIa) (Fig. 2).

### Sample preparation, DNA extraction and polymerase chain reaction methods

Small mammal species identification and dissection were conducted as described previously [27, 28]. Ticks were identified to species level using morphological characters in accordance with Siuda [29] and Estrada-Peña et al. [30]. Before DNA extraction, the ticks were washed twice in distilled water and phosphate-buffered saline and then air dried. DNA extraction of ticks and small mammal skin samples was carried out for each sample individually using the QIAamp DNA Mini Kit (Qiagen, Hilden, Germany) [31]. The quality and quantity of the DNA samples were measured with a spectrophotometer (NanoDrop 2000c; Peqlab Biotechnologie, Erlangen, Germany). The extracted DNA was stored at − 20 °C until further examination.

For the detection of *Borrelia* spp. DNA, small mammal and tick samples were first screened by real-time polymerase chain reaction (PCR) targeting the p41 flagellin gene with an expected amplicon of 96 base pairs [32]. In order to identify the *Borrelia* genospecies and STs of positive samples yielding a cycle threshold (Ct) value below 35, a MLST targeting the housekeeping genes *nifS*, *pyrG*, *clpX*, *pepX*, *uvrA*, *rplB*, *cplA*, and *recG* was performed, with slight modifications [14, 23]. In our experience, the amount of pathogen DNA in samples with Ct < 35 is too low for sufficient amplification by conventional PCR. For samples that did not yield PCR products for all eight MLST housekeeping genes and for which the identification of a specific ST was impossible, genospecies determination was based on *recG* gene sequences.

PCR products were visualized under ultraviolet (UV) light using the UVP GelSolo Simplified UV Gel Documentation System (Analytik Jena, Jena, Germany). For samples positive for all eight PCRs, all the housekeeping genes were sequenced (Eurofins Genomics, Ebersberg, Germany), with forward and reverse primers of each gene used for PCR amplification. The sequence results were analysed with Bionumerics software (version 7.6.1.; Applied Maths, Austin, TX), and compared with sequences published in GenBank using BLASTn (https://blast.ncbi.nlm.nih.gov/Blast.cgi). The aligned sequences of the eight housekeeping loci were compared with those in the online MLST database to assign allele and ST profiles. Novel STs were submitted to the curator, who allocated them consecutive numbers (IDs 3255–3267 with respective STs 986–998).

### Statistical analysis

Confidence intervals (CIs; 95% CI) for the prevalences were determined by the Clopper and Pearson method using GraphPad software (Graph Pad Software, San Diego, CA). A chi-square test was used to test the independence of *Borrelia* prevalence for the factors Small mammal species, Tick life stage, Season, Habitat, and Collection site (GraphPad Software). To analyse *B. burgdorferi* s.l. prevalence in small mammals in relation to season, habitat, and small mammal species, we used a generalized linear mixed model (GLMM) with binomial error distribution using R software (version 4.1.2. for Windows; RStudio, Boston, MA) and the lme4 package [33]. Infection status was used as a binary dependent variable (*Borrelia* spp. positive = 1; *Borrelia* spp. negative = 0). The GLMM was generated to estimate how (i) seasonality (independent binary variable: summer vs. spring), (ii) small mammal species (independent categorical variable), and (iii) habitat (independent binary variable: forest vs. grassland) affect individual infection status (dependent binary variable).

The computed similar approach was used for ticks with the dependent variable *Borrelia* prevalence and the independent variables Tick developmental stage (binary: adult vs. immature), Habitat (see above) and Season (three stages: autumn, spring and summer). *Dermacentor reticulatus* ticks were excluded from this GLMM as none of them was *Borrelia *positive and because the sample size was low.

For small mammals and ticks, the interaction term for the GLMM consisted of three variables with at least two levels each. Therefore, we computed marginal means using the emmeans package within R and a post hoc test to compare the effects of all independent variables separately [34]. Additionally, a generalized linear model (lme4 package in R) was performed with a binary error distribution and a logit link function in order to determine if the number of small mammal species (species richness) per site [35] influences the probability of *Borrelia* infection in individual ticks and small mammals. The significance threshold was set at *P* ≤ 0.05.

## Results

### Small mammal collection

The selected samples (*n* = 1167) were from 10 small mammal species, comprising seven rodent and three shrew species (Table 1). The most common species was the common vole (*Microtus arvalis*) (*n* = 407; 34.9%), followed by the bank vole (*Clethrionomys glareolus*) (*n* = 278; 23.8%) and the yellow-necked field mouse (*Apodemus flavicollis*) (*n* = 240; 20.6%); next were the wood mouse (*Apodemus sylvaticus*) (*n* = 108; 9.3%) and the striped field mouse (*Apodemus agrarius*) (*n* = 90; 7.7%). The common shrew (*Sorex araneus*) (*n* = 20; 1.7%), the field vole (*Microtus agrestis*) (*n* = 15; 1.3%), the water vole (*Arvicola amphibius*) (*n* = 4; 0.3%), the pygmy shrew (*Sorex minutus*) (*n* = 4; 0.3%), and the greater white-toothed shrew (*Crocidura russula*) (*n* = 1; 0.1%) were rare. The three least abundant species were excluded from the analyses due to their low numbers. Altogether, 504 (43.2%) of the small mammals were from grasslands and 663 (56.8%) from forests (Table 1). In the grasslands, the predominant species was *M. arvalis* (74.8%) and in the forests there were two dominant species, *C. glareolus* (40.1%) and *A. flavicollis* (33.2%). The majority of individuals were caught in the summer (*n* = 999; 85.6%); fewer of the small mammals were collected in the spring (*n* = 168; 14.4%).

**Table 1:** Numbers of tested individuals per small mammal species and habitat, and in total

Small mammal species	Number of individuals {*n* (%), [median (range)]}		
	Complete site (part I and II)	Grassland (I)	Forest (II)
*Apodemus agrarius* (striped field mouse)	90 (7.7%), [1 (0–37)]	46 (9.1%), [0 (0–21)]	44 (6.6%), [0 (0–16)]
*Apodemus flavicollis* (yellow-necked field mouse)	240 (20.6%), [11 (0–24)]	20 (4%), [0 (0–6)]	220 (33.2%), [10 (0–22)]
*Apodemus sylvaticus* (wood mouse)	108 (9.3%), [4 (0–26)]	28 (5.6%), [0 (0–9)]	80 (12.1%), [3 (0–17)]
*Arvicola amphibius* (water vole)	4 (0.3%), [0 (0–2)]	3 (0.6%), [0 (0–2)]	1 (0.2%), [0 (0–1)]
*Clethrionomys glareolus* (bank vole)	278 (23.8%), [12 (0–36)]	8 (1.6%), [0 (0–4)]	270 (40.1%), [11 (0–36)]
*Crocidura russula* (greater white-toothed shrew)	1 (0.1%), [0 (0–1)]	1 (0.2%), [0 (0–1)]	0
*Microtus agrestis* (field vole)	15 (1.3%), [0 (0–6)]	8 (1.6%), [0 (0–6)]	7 (1.1%), [0 (0–3)]
*Microtus arvalis* (common vole)	407 (34.9%), [11 (0–75)]	377 (74.8%), [11 (0–68)]	30 (4.5%), [0 (0–10)]
*Sorex araneus* (common shrew)	20 (1.7%), [0 (0–5)]	11 (2.2%), [0 (0–3)]	9 (1.4%), [0 (0–4)]
*Sorex minutus* (pygmy shrew)	4 (0.3%), [0 (0–2)]	2 (0.4%), [0 (0–2)]	2 (0.3%), [0 (0–1)]
Total	1167 (100%), [45 (3–129)]	504 (43.2%), [15 (0–85)]	663 (56.8%), [25 (3–70)]

### Tick collection

In total, 1115 ticks were collected, but 21 individuals were excluded from further examination due to poor conservation of the material. The remaining 1094 ticks belonged to three species, the predominant *I. ricinus* (*n* = 998; 91.2%), followed by *D. reticulatus* (*n* = 80; 7.3%) and *I. inopinatus* (*n* = 16; 1.5%). For further analysis, data were combined for *I. ricinus* and *I. inopinatus*, and are henceforth referred to as those for ‘*I. ricinus* complex’ [36]. In general, nymphs were the most abundant life stage found (Table 2). Overall, almost twice as many ticks were collected from forests (*n* = 717; 65.5%) compared to grasslands (*n* = 377; 34.5%). However, almost all *D. reticulatus* ticks were found in grasslands (74/80; 92.5%). Most of the ticks were collected in spring (*n* = 822, 75.1%; *I. ricinus* complex, *n* = 785, 77.4%; *D. reticulatus*, *n* = 37, 46.3%), followed by summer (*n* = 203, 18.6%; *I. ricinus* complex, *n* = 202, 19.9%; *D. reticulatus*, *n* = 1, 1.3%), and fall (*n* = 69, 6.3%; *I. ricinus* complex, *n* = 27, 2.7%; *D. reticulatus*, *n* = 42, 52.5%).

**Table 2:** Collected ticks

Tick species	Number of collected individuals {*n* (%), [median (range)]}			
	Total	Females	Males	Nymphs
*Ixodes ricinus* complex^a^	1014 (100%), [34 (2–343)]	75 (7.4%), [3 (1–10)]	111 (10.9%), [2 (0–23)]	828 (81.7%), [22 (1–310)]
*Dermacentor* *reticulatus*	80 (100%), [0 (0–69)]	51 (63.8%), [0 (0–48)]	29 (36.2%), [0 (0–21)]	0
Total	1094 (100%), [42 (12–343)]	126 (11.7%), [4 (1–49)]	140 (12.7%), [4 (0–23)]	828 (75.6%), [22 (1–320)]

^a^*Ixodes ricinus* and *Ixodes inopinatus*

### *Borrelia* spp. prevalence and genospecies in small mammals

*Borrelia* spp. DNA was detected in eight out of the 10 small mammal species, with an overall prevalence of 6.2% (*n* = 72; 95% CI 4.9–7.7) (Table 3). Among the small mammal genera, prevalence was lower in *Apodemus* compared to *Microtus*, *Clethrionomys* and *Sorex* (χ^2^ = 29.122, *df* = 3, *P* < 0.001). *Borrelia* prevalence did not differ between spring and summer (see GLMM in Additional file 1: Table S1). The individual infection probability was not affected by the small mammal species richness per site (Additional file 1: Table S2). The GLMM showed that there were no differences in prevalence between small mammals from grassland (I) and forest (II) (*P* = 0.795), and the bias in prevalence must have been confounded by infection rates per small mammal species, as the small mammal species composition differed between grassland (I) and forest (II), with *M. arvalis* having a high prevalence (9.6%) and being the most abundant small mammal species in the grassland (I), while *A. flavicollis* (prevalence 1.7%) was mostly present in the forest (II) (*P* = 0.478). In general, prevalence differed among sites, and ranged from 0 to 20.5% (χ^2^ = 68.392, *df* = 20, *P* < 0.001). This was due to differences in prevalence among grassland sites (I) (0–30.8%; χ^2^ = 59.381, *df* = 19, *P* < 0.001), while in forest sites (II) there was less difference between prevalences (0–13%; χ^2^ = 27.883, *df* = 20, *P* = 0.112).

**Table 3:** *Borrelia* prevalence in small mammals per habitat type and in ticks per grassland and forested parts of the forest habitat, and in total

Small mammal species	Prevalence of *Borrelia* [% (95% CI), *n* positive/*n* tested]		
	Total	Grassland (I)	Forest (II)
*Apodemus agrarius* (striped field mouse)	1.1% (< 0.01–6.6), 1/90	2.2% (< 0.01–12.4), 1/46	0/44
*Apodemus flavicollis* (yellow-necked field mouse)	1.7% (0.5–4.4), 4/240	5% (< 0.01–25.4), 1/20	1.4% (0.3–4.1), 3/220
*Apodemus sylvaticus* (wood mouse)	1.9% (0.1–6.9), 2/108	3.6% (< 0.01–19.2), 1/28	1.3% (< 0.01–7.4), 1/80
*Arvicola amphibius* (water vole)	25% (35.1–71.1), 1/4	33.3% (5.6–79.8), 1/3	0/1
*Clethrionomys glareolus* (bank vole)	7.2% (4.7–10.9), 20/278	12.5% (0.1–49.2), 1/8	7% (4.5–10.8), 19/270
*Crocidura russula* (greater white-toothed shrew)	0/1	0/1	0
*Microtus agrestis* (field vole)	6.7% (< 0.01–31.8), 1/15	12.5% (0.1–49.2), 1/8	0/7
*Microtus arvalis* (common vole)	9.6% (7.1–1.9), 39/407	9% (6.5–12.4), 34/377	16.7% (6.9–34), 5/30
*Sorex araneus* (common shrew)	20% (7.5–42.2), 4/20	18.2% (4–48.9), 2/11	22.2% (5.3–55.7), 2/9
*Sorex minutus* (pygmy shrew)	0/4	0/2	0/2
Total	6.2% (4.9–7.7), 72/1167	8.3% (6.2–11.1), 42/504	4.5% (3.2–6.4), 30/663

Tick species	Total	Grassland part of forest (IIb)	Forest part of forest (IIa)
*Ixodes ricinus* complex	13% (11.1–15.2), 132/1014	16.8% (13–21.5), 51/303	11.4% (9.3–14), 81/711
*Dermacentor reticulatus*	0/80	0/74	0/6
Total	12.1% (10.3–14.1), 132/1094	13.5% (10.4–17.4), 51/377	11.3% (9.2–13.9), 81/717

*CI* Confidence interval

Out of 72 positive samples identified by real-time PCR, eight yielded a Ct value < 35 and were sequenced (one from *Cl glareolus*, four from *M. arvalis* and three from *S. araneus*). The analysis revealed the presence of two genospecies (Additional file 1: Table S5; Table 4): *B. afzelii* in seven individuals of three host species, and *B. garinii* in one *M. arvalis*. The *recG* gene sequences showed 100% identity to existing GenBank entries (Table 4).

**Table 4:** Sequencing results based on the *recG* gene in tick and small mammal samples that did not yield multilocus sequence typing products

Sample				*Borrelia* genospecies	Most similar sequence	
Sample identifier (ID)	Host species	Collection site	Habitat		Identity (%)	GenBank ID
HT1	*Ixodes ricinus*	UH18	Grassland	*Borrelia spielmanii*	100	AB526160
HT7	*I. ricinus*	UH18	Grassland	*Borrelia valaisiana*	100	CP009117
HT15	*I. ricinus*	UH18	Grassland	*Borrelia afzelii*	99.7	CP000395
HT20	*I. ricinus*	UH18	Forest	*B. spielmanii*	99	AB526160
HT32	*I. ricinus*	UH16	Forest	*B. afzelii*	100	JX971362
HT38	*I. ricinus*	UH16	Forest	*B. afzelii*	99.9	CP009058
HT68	*I. ricinus*	Kyf2	Forest	*B. valaisiana*	99.9	CP009117
HT81	*I. ricinus*	E3	Grassland	*B. afzelii*	100	JX971362
HT99	*I. ricinus*	E3	Grassland	*Borrelia garinii*	99.1	JF331209
HT115	*I. ricinus*	E3	Forest	*B. valaisiana*	100	CP009117
HT126	*I. ricinus*	E1	Grassland	*Borrelia lusitaniae*	100	MH747538
HT192	*I. ricinus*	E1	Forest	*B. garinii*	100	JF331209
HT199	*I. ricinus*	E1	Forest	*B. afzelii*	99.9	JX971362
HT217	*I. ricinus*	E1	Forest	*B. lusitaniae*	99.7	MH747538
HT261	*I. ricinus*	UH7	Grassland	*Borrelia burgdorferi* s.s.	98.6	JF419041
HT298	*I. ricinus*	UH9	Grassland	*B. afzelii*	97.1	JX971362
HT303	*I. ricinus*	UH9	Grassland	*B. garinii*	100	JF331209
HT307	*I. ricinus*	UH9	Grassland	*B. garinii*	100	AB555923
HT310	*I. ricinus*	UH9	Grassland	*B. afzelii*	97.1	JX971362
HT324	*I. ricinus*	UH9	Forest	*B. valaisiana*	99.7	CP009117
HT331	*I. ricinus*	UH9	Forest	*B. afzelii*	100	JX971362
HT332	*I. ricinus*	UH9	Forest	*B. afzelii*	99.9	CP002933
HT375	*I. ricinus*	UH1	Grassland	*B. valaisiana*	99.3	MG972813
KS18/1496	*Sorex araneus*	UH12	Grassland	*B. afzelii*	100	MG972813
KS19/1709	*Microtus arvalis*	UH16	Grassland	*B. garinii*	100	CP028861
KS19/2049	*M. arvalis*	UH1	Grassland	*B. afzelii*	100	CP018262

### *Borrelia* spp. prevalence and genospecies in ticks

*Borrelia* spp. DNA was detected in 132 out of 1094 ticks (12.1%; 95% CI 10.3–14.1). All of the positive samples originated from *I. ricinus* complex ticks (*n* = 1014) (13%; 95% CI 11.1–15.2). The prevalence was higher in females (19/75, 25.3%; 95% CI 16.8–36.3) than in males (19/111, 17.1%; 95% CI 11.2–25.3), and lowest in nymphs (94/828, 11.4%; 95% CI 9.4–14.5) (χ^2^ = 13.721, *df* = 2, *P* = 0.001). *Ixodes ricinus* complex ticks collected in the grasslands (IIb) were significantly more often infected (51/303, 16.8%; 95% CI 13–21.5) than those from forests (IIa) (81/711, 11.4%; 95% CI 9.3–14) (see GLMM in Additional file 1: Table S3). More detailed analyses revealed that a higher prevalence in adults compared to nymphs was true for grasslands (*P* = 0.015) but not for forests (*P* = 0.849) (Additional file 1: Table S3). The GLMM also revealed that seasonal fluctuation of prevalence was a confounding factor (*P* = 0.323–0.989) and could be explained by a seasonal effect of the life stage composition of *I. ricinus*. Adults were significantly more often positive than nymphs (*P* = 0.0155), and in spring the ratio of nymphs to adults was 4:1, while in summer it was 13:1. The prevalence among sites varied significantly, from 0 to 28.6% (χ^2^ = 39.013, *df* = 16, *P* = 0.001); it was significant for grasslands (IIb), where the prevalence range was higher (0–75%; χ^2^ = 41.205, *df* = 16; *p* < 0.001), but not for forests (IIa) (0–15.8; χ^2^ = 23.952, *df* = 16, *P* = 0.09). The generalized linear model suggested that the probability of infection in ticks was positively correlated to the number of small mammal species per site (Additional file 1: Table S4).

Out of 132 positive tick samples, 69 *recG* gene PCR products were sequenced; this revealed the presence of six genospecies (Additional file 1: Table S5 and Table 4): *B. afzelii* (*n* = 37), *B. garinii* (*n* = 16), *B. valaisiana* (*n* = 9), *B. lusitaniae* (*n* = 3), *B. burgdorferi* s.s. (*n* = 2), and *B. spielmanii* (*n* = 2). All of the genospecies were detected in ticks collected from grasslands (IIb) and forests (IIa). However, the genospecies composition differed between habitats, with an almost equal genospecies distribution of *B. afzelii* and *B. garinii* (44% vs. 35%) in the grasslands and a 4:1 ratio of *B. afzelii* to *B. garinii* in forests (60% vs. 15%). The *recG* gene sequences showed very high (97.1–100%) identity to GenBank entries of the corresponding reference strains (Table 4).

### MLST analysis

MLST analyses were performed for 51 *Borrelia*-positive samples obtained from 46 *I. ricinus* complex ticks and five small mammals. The MLST results were ambiguous for 16 samples, and thus STs could not be assigned for these. In total, the remaining 35 samples revealed 28 STs, 25 in ticks and three in small mammals, indicating a genetically diverse *Borrelia* population (Additional file 1: Table S5). Only three STs were detected more than once, and all of these were from tick samples. The most common of these was ST 347, belonging to *B. afzelii*, which was found in five ticks from three different sites across both forest habitats (IIa and IIb), followed by ST 251 of *B. garinii*, which was detected in three ticks from two sites and habitats. The third ST that was found multiple times was *B. garinii* ST 187, but only in ticks from two different spots in forests (IIa). The three STs detected in common shrews and a common vole were not found in ticks (Additional file 1: Table S5). In grasslands, 10 different STs were found in ticks (IIb) and two in small mammals (I). In forest sites, 17 STs were identified in ticks (IIa) and one in small mammals (II). The highest diversity of STs was noted for *B. afzelii* (*n* = 16), followed by *B. garinii* (*n* = 9), *B. valaisiana* (*n* = 2) and *B. burgdorferi* s.s. (*n* = 1). No STs were assigned for *B. spielmanii* or *B. lusitaniae*.

## Discussion

Small mammals are important reservoir hosts for *B. burgdorferi* s.l. [4, 12, 13, 37, 38]. In our study, 10 small mammal species were examined, almost all of which are recognized reservoir hosts of *B. burgdorferi* [4, 5, 12, 13]*. Borrelia* spp. DNA was found in eight out of the 10 species, but not in *C. russula* and *S. minutus*, most probably due to their low sample size. However, these two shrew species were found to be *Borrelia* positive in previous studies [4, 39].

The mean prevalence in small mammals in this study was 6.2%, which is in line with the results of previous European studies that showed prevalences of 1.2–24% [40–46]. However, it was lower than in other studies with a lower number of small mammal species (31.2–49.1%) [14, 31]. Although *Apodemus* had moderate to high prevalence (e.g. 25.4–47.5%) in other studies [14, 31], in our study the lowest *Borrelia* spp. prevalence was in this genus. There was no seasonal pattern for *Borrelia* prevalence in small mammals, most probably because the samples derived only from spring and summer sampling. A similar absence of a seasonal pattern was also observed in a study from Spain [39].

*Dermacentor reticulatus* ticks have been found to carry *Borrelia* in Germany [47, 48], but *Borrelia*-negative *D. reticulatus* ticks have also been reported in several studies from Germany [14, 31, 49–51]. In our study, none of the *D. reticulatus* ticks (*n* = 80) was positive for *B. burgdorferi* s.l., although at two spots *Borrelia* DNA was detected in sympatric *I. ricinus* complex ticks. These findings support the view that *D. reticulatus* is of minor importance in the natural life cycle of the *B. burgdorferi* species complex [3].

*Borrelia* prevalence in the *I. ricinus* complex in the current study was 13%, which is in agreement with the results of European meta-analyses from the last decades, i.e. 13.7% (1984–2003) and 12.3% (2010–2016) [52, 53]. The level of infection was higher in adults (20.4%) than in nymphs (11.1%), as also found in other studies (18.6 vs. 10.1% [52]; and 14.9 vs. 11.8% [53]), and is a result of *Borrelia* transstadial transmission and a higher number of blood meals on reservoir hosts for adults. As seasonality was not an important explanatory variable in the GLMM analysis, the lack of significant differences in prevalence between the seasons could be explained by the different seasonal ratios and activity levels of immature and adult ticks.

*Borrelia* prevalence in ticks and small mammals differed significantly among grassland sites, which suggests that local factors may impact the maintenance of pathogen circulation. Such factors could be land use that influences the abundance of the potential hosts—not only small but also medium- and large-sized mammals, as well as birds. Interestingly, the prevalence was lower in ticks from forests (IIa) than from grasslands (IIb), possibly because of a higher diversity of wildlife in forests [54, 55].

Our analysis showed that *Borrelia* spp. prevalence in ticks is positively correlated to the number of small mammal species per site: the more reservoir host species, the higher the infection probability in ticks. This seems to be in line with the dilution effect hypothesis [56, 57], which assumes that reservoir competence should vary between host species. In addition, in our study, almost all of the tested animals were reservoir hosts (e.g. [4]) with a different prevalence of *Borrelia*; therefore, an amplification effect was observed, meaning the more reservoir species the higher the prevalence in vectors. The number of small mammal species per site did not influence *Borrelia* prevalence in the hosts themselves.

Six *Borrelia* genospecies were identified in the present study. *B. afzelii* was predominant in ticks (37/69) and small mammals (7/8), followed by *B. garinii* [which was found in ticks (16/69) and small mammals (1/8)]; similar results have been previously reported [31, 58] for Germany and elsewhere in Europe [53]. The other genospecies—*B. valaisiana* (6/69), *B. lusitaniae* (3/69), *B. burgdorferi* s.s. (2/69) and *B. spielmanii* (2/69)—were found only in ticks, and have been described before in ticks from Germany at similar frequencies [59, 60]. The diversity of the detected genospecies was higher [14, 31, 58] or similar to that of other studies carried out in Germany, Belarus, and Romania [59–62]. The reason for this might be the association of certain genospecies with the abundance of their respective reservoirs. Rodents are considered the main reservoir hosts for following *Borrelia* genospecies: *B. afzelii*, *B. lusitaniae*, *B. burgdorferi* s.s. and *B. spielmanii*, while small birds for *B. garinii*, *B. valaisiana* as well as *B. burgdorferi* s.s. Some years ago, *B. garinii* OspA serotype 4 strains were reclassified as *B. bavariensis*, as they use rodents as reservoir hosts instead of birds [11, 63–65]. However, as we did not sequence *ospA*, which could have been used to discriminate the genospecies, we could only compare the sample from *M. arvalis* (KS19/1709) to GenBank data; our *recG* sequence was a 100% match to *B. garinii* (GenBank no. CP028861). Additionally, lizards are recognized reservoirs for *B. lusitaniae*, *B. valaisiana* and *B. garinii*, and larger mammals for *B. burgdorferi* s.s.

MLST has been performed for *Borrelia* spp. genotyping in ticks from 21 European countries, but for vertebrate hosts only for six European countries (pubmlst.org, accessed on 1 Feb 2022). Our MLST analyses for tick- and small mammal-derived samples revealed 15 different STs that have been previously published. Most of these STs were previously detected in humans and/or ticks from European countries. Six of these STs have been reported in ticks from eight European countries, but never from Germany. One ST (ST 467) that has not been previously reported in ticks was previously reported in a person from France [66]. ST 338, which was detected in a small mammal in the current study, has been formerly described in ticks from Austria (pubmlst.org). The other ST found in the small mammals (ST 348) has been previously described from a person from Germany, but has never previously been reported for other animals. Additionally, we discovered 13 new STs that have never been reported before (STs 986–998): 12 from ticks and one from a shrew. The STs found in the ticks did not overlap with those detected in the shrews and voles, suggesting that the ticks acquired the pathogens from other reservoir hosts. However, it should be borne in mind that the number of STs determined in the small mammals was very low. This high variety of STs (at least 28) indicates a very diverse *Borrelia* population, even at individual sites (e.g. E1, E3, UH1). The quantity and variety of *Borrelia* STs reflected the generally high plant and animal biodiversity of this region, which has many potential host species [20, 57, 67–69]. However, as we tested only small mammals, other potential host groups should be investigated in future research.

## Conclusions

This study was conducted in an area of high biodiversity with numerous species of animals and plants. Our results showed a high species richness of small mammals (*n* = 10), ticks (*n* = 3) and *Borrelia* genospecies (*n* = 6). Likewise, the number of detected *Borrelia* STs (≥ 28) was also very high. The genetic variation of *B. afzelii* in ticks was not aligned with that in shrews and voles, which points to other vertebrate host species contributing to the life cycle of this pathogen. The prevalence of *Borrelia* in ticks seemed to be influenced by habitat, as in grassland habitats adjacent to forest the prevalence was higher than in the forested habitats. Even though the prevalence of *Borrelia* in ticks and small mammals was moderate, all the genospecies detected have zoonotic potential and constitute a disease risk for humans. Therefore, the attention of public health authorities should be drawn to the results presented here for this region of Germany.

## Supplementary Information

**Additional file 1: Table S1**. Results of a GLMM with a binomial error distribution for effects of habitat, small mammal species and season on *Borrelia *spp. infection probability in small mammals. **Table S2**. Generalized linear model for *Borrelia *spp. infection probability in small mammals according to the number of small mammal species per site. **Table S3**. Results of a GLMM with a binomial error distribution for effects of habitat, tick developmental stage and seasonality on *Borrelia *spp. infection probability in ticks. **Table S4**. Generalized linear model results for *Borrelia *spp. infection probability in ticks according to the number of small mammal species detected per site. **Table S5**. Results of MLST analyses of *Borrelia *spp. samples from ticks and small mammals.

## Data Availability

The datasets generated and analysed during the current study are available from the corresponding author.

## Abbreviations

CI: Confidence interval
Ct: Cycle threshold
GLMM: Generalized linear mixed model
MLST: Multilocus sequence typing
PCR: Polymerase chain reaction
s.l.: Sensu lato
s.s.: Sensu stricto
STs: Sequence types
