# Supplementary material for: Diversity of Borrelia burgdorferi sensu lato in ticks and small mammals from different habitats
Source: Parasit Vectors. 2022 Jun 7;15:195. doi: 10.1186/s13071-022-05326-3 (PMC9175456; doi:10.1186/s13071-022-05326-3)
Supplement: Supplementary file 1 — Additional file 1: Table S1. Results of a GLMM with a binomial error distribution for effects of habitat, small mammal species and season on Borrelia spp. infection probability in small mammals. Table S2. Generalized linear model for Borrelia spp. infection probability in small mammals according to the number of small mammal species per site. Table S3. Results of a GLMM with a binomial error distribution for effects of habitat, tick developmental stage and seasonality on Borrelia spp. infection probability in ticks. Table S4. Generalized linear model results for Borrelia spp. infection probability in ticks according to the number of small mammal species detected per site. Table S5. Results of MLST analyses of Borrelia spp. samples from ticks and small mammals. [file 13071_2022_5326_MOESM1_ESM.pdf]

Supplementary Table 1. Results of a generalized linear mixed model with binomial error distribution for effects of habitat, small mammal species and season on *Borrelia* spp. infection probability in small mammals.

	Estimate	Standard error	Z value	Probability (>|z|)
**Total**				
(Intercept)	4.393	-1.1136	-3.945	7.98e-05 ***
Spring - Summer	-0.2705	0.3815	-0.709	0.4783
Grass – Wood	-0.1235	0.4749	-0.26	0.7948
A. agrarius - A. flavicollis	0.2172	1.1458	0.19	0.8497
A. agrarius - A. sylvaticus	0.4998	1.2416	0.403	0.6873
A. agrarius - M.agrestis	1.9177	1.4536	1.319	0.1871
A. agrarius - M.arvalis	1.9913	1.0481	1.9	0.0574 .
A. agrarius - Cl. glareolus	1.8648	1.07	1.743	0.0814 .
A. agrarius - S. araneus	2.9765	1.1739	2.535	0.0112 *
**Grassland**				
(Intercept)	3.8288	1.2721	-3.01	0.00261 **
Spring - Summer	-0.2143	0.5624	-0.381	0.70309
A. agrarius - A. flavicollis	0.529	1.5274	0.346	0.72907
A. agrarius - A. sylvaticus	0.7458	1.559	0.478	0.63236
A. agrarius - M.agrestis	2.3414	1.6825	1.392	0.16405
A. agrarius - M.arvalis	0.9887	1.1479	0.861	0.38906
A. agrarius - Cl. glareolus	2.3176	1.6398	1.413	0.15755
A. agrarius - S. araneus	2.0528	1.4259	1.440	0.14995
**Forest**				
(Intercept)	-19.63284	41.68606	-0.007	0.638
A. agrarius - A. flavicollis	15.22931	41.68927	0.365	0.715
A. agrarius - A. sylvaticus	15.18204	41.69927	0.364	0.716
A. agrarius - M.agrestis	-0.09782	170.88751	-0.001	1.000
A. agrarius - M.arvalis	17.86194	41.68867	0.428	0.668
A. agrarius - Cl. glareolus	16.97026	41.68625	0.407	0.684
A. agrarius - S. araneus	18.13702	41.69365	0.435	0.664

Significance: "***" - <0.001; "**" - 0.001; "*" - 0.01; "." - 0.05

Supplementary Table 2. Generalized linear model for *Borrelia* spp. infection probability in small mammals according to the number of small mammal species per site.

	Estimate	Standard error	Z value	Probability (>|z|)
(Intercept)	-2.5634	0.39737	-6.451	1.11e-10 ***
Small mammal species number	-0.02744	0.08005	-0.343	0.732
**Log model coefficients**				
(Intercept)	-2.56340049	0.39737364	-6.450857	1.112195e-10
Small mammal species number	-0.02744081	0.08004646	-0.342811	7.317406e-01

Significance: "***" - <0.001

Supplementary Table 3. Results of a generalized linear mixed model with binomial error distribution for effects of habitat, tick developmental stage and seasonality on *Borrelia* spp. infection probability in ticks.

Total				
	**Estimate**	**Standard error**	**Z value**	**Probability (>|z|)**
(Intercept)	-2.203254	0.663254	-3.322	0.000894 ***
Autumn - Spring	0.624191	0.63201	0.988	0.323335
Autumn - Summer	0.008619	0.664343	0.013	0.989648
Grass - Wood	0.430987	0.203598	-2.117	0.034273 *
Adults - Nymphs	-0.5457	0.2256	-2.419	0.0155 *
**Grassland**				
(Intercept)	-16.7049	223.4572	-0.075	0.9404
Autumn - Spring	15.6298	223.4576	0.07	0.9442
Autumn - Summer	15.2969	223.4555	0.068	0.9454
Adults - Nymphs	-0.8197	0.3366	-2.435	0.0149 *
**Forest**				
(Intercept)	-2.28425	0.74981	-3.046	0.00232 **
Autumn - Spring	0.27626	0.65517	0.422	0.67327
Autumn - Summer	-0.27017	0.68405	-0.395	0.69288
Adults - Nymphs	-0.06565	0.34596	-0.190	0.84950

Significance: “***” - <0.001; “**§ - 0.001; “*” - 0.01

Supplementary Table 4. Generalized linear model results for *Borrelia* spp. infection probability in ticks according to the number of small mammal species detected per site.

	**Estimate**	**Standard error**	**Z value**	**Probability (>|z|)**
(Intercept)	-2.91019	0.36875	-7.892	2.97e-15 ***
Small mammal species number	0.22177	0.07427	2.986	0.00283 **
**Log model coefficients**				
(Intercept)	-2.9101874	0.36874565	-7.892127	2.970802e-15
Small mammal species number	0.2217718	0.07427471	2.985832	2.828084e-03

Significance: “***” - <0.001; “**” - 0.001

Supplementary Table 5. Results of MLST analyses of *Borrelia* spp. samples from ticks and small mammals.

Sample				Number of sequence type								ST	*Borrelia* genospecies
Sample ID	Host species	Collection site	Habitat	*clpA*	*clpX*	*nifS*	*pepX*	*pyrG*	*recG*	*rplB*	*uvrA*		
HT5	*I. ricinus*	UH18	grassland	38	24	79	31	-	75	-	76	-	*B. afzelii*
HT10	*I. ricinus*	UH18	grassland	95	-	34	96	89	78	32	85	251	*B. garinii*
HT19	*I. ricinus*	UH18	grassland	109	24	23	31	-	27	-	28	-	*B. afzelii*
HT23	*I. ricinus*	UH17	grassland	36	24	23	85	23	27	23	30	995	*B. afzelii*
HT29	*I. ricinus*	UH16	grassland	114	24	24	31	22	92	23	28	347	*B. afzelii*
HT85	*I. ricinus*	E3	grassland	42	27	29	38	29	36	27	33	86	*B. garinii*
HT99	*I. ricinus*	E3	grassland	46	76	29	43	34	40	31	37	743	*B. garinii*
HT107	*I. ricinus*	E3	forest	95	74	34	96	89	78	77	85	251	*B. garinii*
HT111	*I. ricinus*	E3	forest	49	37	37	45	39	45	36	40	99	*B. valaisiana*
HT133	*I. ricinus*	E1	grassland	114	24	24	31	-	30	-	28	-	*B. afzelii*
HT145	*I. ricinus*	E1	grassland	114	24	24	31	22	92	23	28	347	*B. afzelii*
HT157	*I. ricinus*	E1	grassland	37	24	24	31	-	28	23	-	-	*B. afzelii*
HT166	*I. ricinus*	E1	forest	109	24	96	31	-	30	23	29	-	*B. afzelii*
HT178	*I. ricinus*	E1	forest	35	24	96	31	22	172	23	28	993	*B. afzelii*
HT364	*I. ricinus*	UH1	forest	114	24	24	31	22	92	23	28	347	*B. afzelii*
HT365	*I. ricinus*	UH1	forest	47	73	33	42	91	76	32	36	187	*B. garinii*
HT367	*I. ricinus*	UH1	forest	50	36	36	45	38	44	35	40	97	*B. valaisiana*
HT370	*I. ricinus*	UH1	forest	-	24	24	85	-	91	35	29	-	*B. afzelii*
HT380	*I. ricinus*	UH1	forest	114	24	24	31	22	92	23	28	347	*B. afzelii*
HT569	*I. ricinus*	UH18	forest	109	24	23	31	119	27	23	28	-	*B. afzelii*
HT628	*I. ricinus*	E3	grassland	95	74	34	96	89	78	77	85	251	*B. garinii*
HT632	*I. ricinus*	E3	grassland	50	36	36	45	234	44	35	35	-	*B. valaisiana*
HT633	*I. ricinus*	E3	grassland	44	36	31	45	38	37	35	35	-	*B. garinii*
HT635	*I. ricinus*	E3	forest	47	73	33	42	91	76	32	36	187	*B. garinii*
HT654	*I. ricinus*	E1	grassland	51	24	24	85	22	91	24	29	988	*B. afzelii*
HT712	*I. ricinus*	E1	forest	109	24	23	89	96	27	23	28	467	*B. afzelii*
HT717	*I. ricinus*	E1	forest	183	24	24	31	22	172	23	28	-	*B. afzelii*
HT736	*I. ricinus*	E1	forest	114	24	96	31	119	29	23	28	992	*B. afzelii*
HT745	*I. ricinus*	E1	forest	114	24	24	31	272	92	23	28	-	*B. afzelii*
HT749	*I. ricinus*	E1	forest	114	24	24	31	272	92	23	28	-	*B. afzelii*
HT752	*I. ricinus*	E1	forest	36	82	24	31	85	29	23	28	998	*B. afzelii*
HT765	*I. ricinus*	E1	forest	183	24	24	85	22	91	23	29	778	*B. afzelii*
HT768	*I. ricinus*	E1	forest	114	24	24	31	22	92	23	28	347	*B. afzelii*
HT796	*I. ricinus*	E1	forest	114	24	96	31	22	92	23	28	989	*B. afzelii*
HT806	*I. ricinus*	E1	forest	109	82	185	31	85	30	23	28	996	*B. afzelii*
HT875	*I. ricinus*	UH7	grassland	47	32	33	42	35	88	32	36	189	*B. garinii*
HT889	*I. ricinus*	UH7	grassland	109	24	96	31	85	172	23	28	994	*B. afzelii*

HT957	*I. ricinus*	UH18	forest	114	24	24	31	98	92	23	28	-	*B. afzelii*
HT968	*I. ricinus*	Kyf1	forest	47	73	81	42	91	76	32	36	986	*B. garinii*
HT993	*I. ricinus*	E1	grassland	99	77	81	91	88	84	82	33	245	*B. garinii*
HT1041	*I. ricinus*	UH1	grassland	167	131	120	273	144	144	126	136	970	*B. garinii*
HT1063	*I. ricinus*	UH1	forest	51	24	24	31	22	27	23	28	991	*B. afzelii*
HT1068	*I. ricinus*	UH1	forest	114	24	14	31	22	92	23	28	990	*B. afzelii*
HT1071	*I. ricinus*	UH1	forest	17	10	14	10	10	13	9	17	27	*B. burgdorferi* s.s.
HT1078	*I. ricinus*	UH1	forest	114	24	185	31	272	92	23	28	-	*B. afzelii*
HT1096	*I. ricinus*	W1	forest	45	30	32	41	32	38	30	36	90	*B. garinii*
KS18/1460	*S. araneus*	UH16	forest	114	24	24	31	116	92	23	28	338	*B. afzelii*
KS18/1521	*S. araneus*	UH14	grassland	109	24	96	85	96	27	23	28	987	*B. afzelii*
KS18/1524	*M. arvalis*	UH14	grassland	36	24	23	31	20	28	23	29	348	*B. afzelii*
KS19/1655	*M. arvalis*	E1	grassland	-	24	23	31	92	172	24	28	-	*B. afzelii*
KS19/2451	*Cl. glareolus*	UH3	grassland	109	212	89	89	-	27	23	-	-	*B. afzelii*

“-“ – no match
